# Testicular activin and follistatin levels are elevated during the course of experimental autoimmune epididymo–orchitis in mice

**DOI:** 10.1038/srep42391

**Published:** 2017-02-13

**Authors:** Nour Nicolas, Vera Michel, Sudhanshu Bhushan, Eva Wahle, Susan Hayward, Helen Ludlow, David M. de Kretser, Kate L. Loveland, Hans-Christian Schuppe, Andreas Meinhardt, Mark P. Hedger, Monika Fijak

**Affiliations:** 1 Department of Anatomy and Cell Biology, Justus-Liebig University, Giessen, Germany; 2 Hudson Institute of Medical Research, Melbourne, Victoria, Australia; 3 Oxford-Brooks University, Oxford, England; 4 Department of Anatomy and Developmental Biology, Monash University, Melbourne, Victoria, Australia; 5 School of Clinical Sciences, Monash University, Melbourne, Victoria, Australia; 6 Department of Urology, Pediatric Urology and Andrology, Justus-Liebig University, Giessen, Germany

## Abstract

Experimental autoimmune epididymo-orchitis (EAEO) is a model of chronic inflammation, induced by immunisation with testicular antigens, which reproduces the pathology of some types of human infertility. Activins A and B regulate spermatogenesis and steroidogenesis, but are also pro-inflammatory, pro-fibrotic cytokines. Expression of the activins and their endogenous antagonists, inhibin and follistatin, was examined in murine EAEO. Adult untreated and adjuvant-treated control mice showed no pathology. All mice immunised with testis antigens developed EAEO by 50 days, characterised by loss of germ cells, immune cell infiltration and fibrosis in the testis, similar to biopsies from human inflamed testis. An increase of total CD45+ leukocytes, comprising CD3+ T cells, CD4 + CD8− and CD4 + CD25+ T cells, and a novel population of CD4 + CD8+ double positive T cells was also detected in EAEO testes. This was accompanied by increased expression of TNF, MCP-1 and IL-10. Activin A and B and follistatin protein levels were elevated in EAEO testes, with peak activin expression during the active phase of the disease, whereas mRNA expression of the inhibin B subunits (*Inha* and *Inhbb*) and activin receptor subunits (*Acvr1b* and *Acvr2b*) were downregulated. These data suggest that activin–follistatin regulation may play a role during the development of EAEO.

Infection and inflammation of the male reproductive tract, including the testis, are important causes of male infertility, as they lead to disruption of spermatogenesis and alteration of sperm number and quality^1^,^2^.

Experimental autoimmune epididymo-orchitis (EAEO) is an established rodent model of chronic testicular inflammation^3^, mimicking the pathology observed in the human testis and resulting in infertility^4^,^5^. From previous studies in rodents, it is known that EAEO is characterised by the production of auto-antibodies against testicular antigens, elevated levels of pro-inflammatory mediators, e.g. monocyte chemoattractant protein-1 (MCP-1), interleukin-6 (IL-6) and tumour necrosis factor (TNF), reduced levels of testosterone in serum and infiltration of the testicular interstitial space with leukocytes such as macrophages, dendritic cells, T cells, all leading to the formation of granulomas^3^,^6^,^7^,^8^,^9^. During the course of EAEO, germ cell sloughing leads to aspermatogenesis and subsequent infertility. However, the exact immunopathological mechanisms of disease development are still unclear and non-invasive tools for early diagnosis as well as possible therapeutic interventions are missing.

Activin A, a member of the transforming growth factor-β (TGFβ) superfamily of cytokines is centrally involved in the control of inflammatory, immune and fibrotic processes. Activin A is formed by a homodimer of two βA subunits linked by a disulphide bond^10^, while a homodimer of two βB subunits forms activin B, which is less biologically active than activin A^11^. These β subunits can also dimerise with an α subunit to form either inhibin A (αβA) or inhibin B (αβB). Inhibins are potent endogenous antagonists of activin A and B, as they block their activity by competing for the activin receptors or by reducing the availability of β subunits to form activin dimers^12^.

Activin A is widely expressed in both reproductive and non-reproductive organs^13^,^14^, with the testis and epididymis being the main sources of activin A in the male reproductive tract^15^.

Similar to the other TGFβ superfamily members, activins bind to a type II receptor (*Acvr2a* or *Acvr2b*), which then recruits the type I receptor, ALK4 (*Acvr1b*) or, in the case of activin B, ALK7 (*Acvr1c*), a serine/threonine kinase, leading to its phosphorylation and the activation of the Smad transcription factor signalling pathway^16^,^17^. Activins can also act via the mitogen-activated protein (MAP) kinase signalling pathway, induced during inflammatory conditions^18^.

In the adult testis, under normal conditions, activin A plays a crucial role in regulation of spermatogenesis and steroidogenesis^14^,^19^. Moreover, activin A is a local regulator of Sertoli and germ cell development, proliferation, differentiation and function^20^,^21^. Furthermore, it has a role in immune regulation and immune cell development, and is implicated in maintaining the immune privileged status of the testis^22^.

Dysregulation of activin A signalling can cause severe adverse effects. Overexpressing activin A in the testis leads to disruption of spermatogenesis and consequently infertility^23^, whereas chronic stimulation of activin signalling causes reduction in testis weight and subsequent hypospermatogenesis^20^. An activin A excess is responsible for promoting fibrosis in many tissues, under pathological conditions^18^,^24^. It has been shown that, in many acute and chronic inflammatory conditions, activin A is systemically elevated^25^,^26^,^27^,^28^. In contrast to activin A, the physiological and pathophysiological roles of activin B in the testis and in inflammation/fibrosis have received little attention.

Follistatin is an endogenous, high affinity activin-binding protein^29^, which blocks activin action by limiting its access to the receptors. By a splicing mechanism of the follistatin gene, *FST*, two forms of follistatin are known to be produced, FST288 and FST315^30^. Due to their differential ability to bind to cell-surface heparin sulphate proteoglycans, FST288 is predominantly the tissue-bound form and FST315 is the main circulating form^31^. Currently, follistatin is being considered as a potential therapeutic target for numerous inflammatory diseases including fibrosis, due to its ability to block activin A actions^32^,^33^,^34^.

Given these findings, and knowing that activin A is increased in a number of inflammatory conditions, we investigated whether the levels of activin A and B and their antagonists, inhibin and follistatin are changed during the course of testicular inflammation and could be involved in disease pathogenesis.

## Results

### Morphological changes in EAEO testes from mice are accompanied by a strong fibrotic response

**Figure 1:**
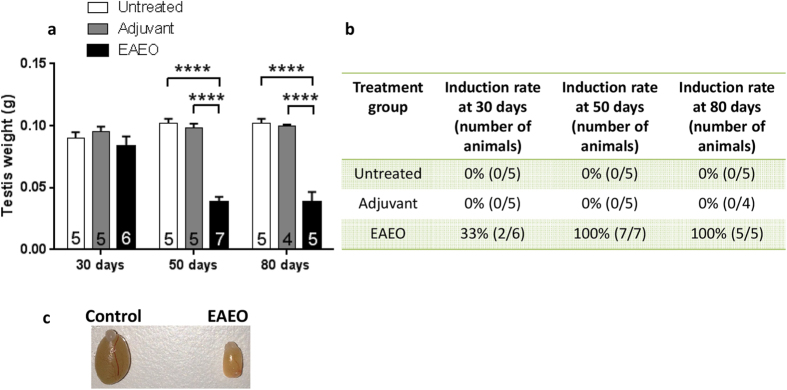
Testicular weight (**a**) and induction rate (**b**) of EAEO in animals used in the study. Paired testicular weight of untreated, adjuvant control and EAEO mice 30, 50 and 80 days after the first immunisation (**a**). Data are expressed as mean ± SEM (n = 4–7 animals per group, numbers of animals per group are shown in the respective columns); ****P < 0.0001, all other comparisons are not statistically significant. Representative image shows macroscopic difference in testicular size from control and EAEO mice 50 days after the first immunisation (**c**).

**Figure 2:**
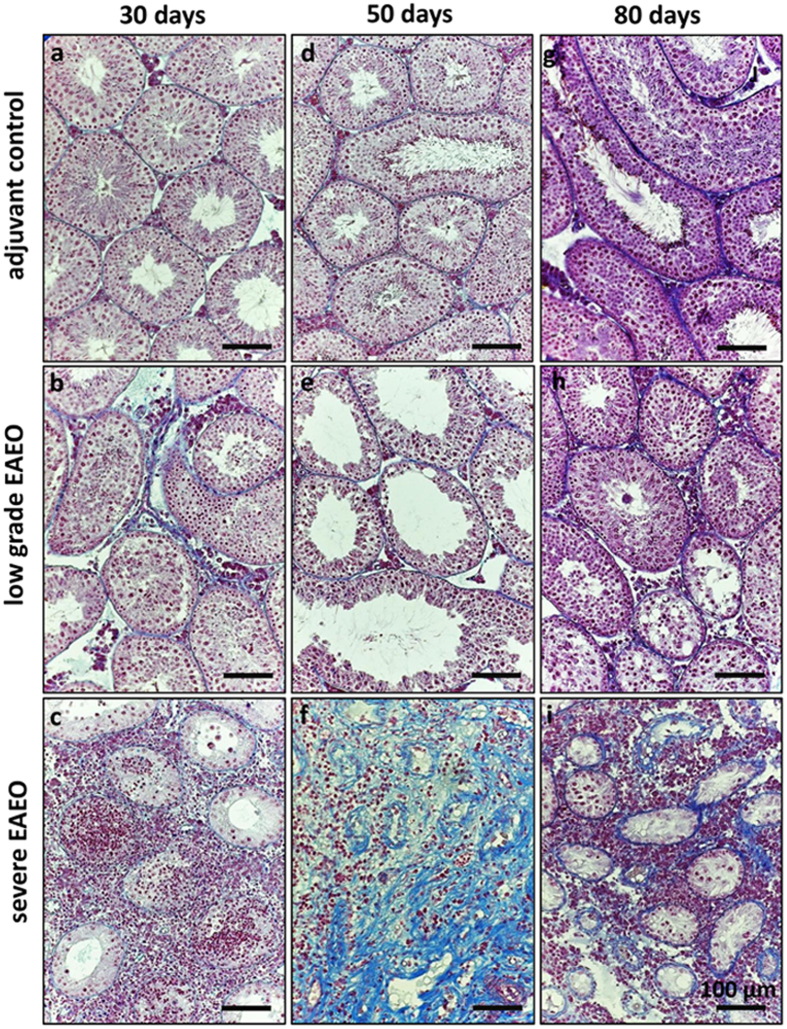
Azo-carmine and aniline blue staining of collagen fibres in paraffin sections from adjuvant control (**a**,**d**,**g**), low grade (**b**,**e**,**h**), and severe EAEO (**c**,**f**,**i**) mouse testes at 30 (**a**–**c**), 50 (**d**–**f**) and 80 (**g**–**i**) days after first immunisation. An increase in collagen fibres is visible in low grade EAEO in the areas with lymphocytic infiltrates. A strong peritubular fibrotic response is detectable in EAEO mouse testes at 50 (**f**) and 80 (**i**) days after the first immunisation. Scale bars represent 100 μm.

Successful induction of EAEO was estimated based on testicular weight and histopathological changes of testicular architecture. In the EAEO group, a more than 2-fold decrease of testis weight collected at 50 (n = 7) and 80 (n = 5) days after the first immunisation compared to untreated (n = 5) and adjuvant control (n = 5 for 50 days or n = 4 for 80 days) groups was observed (Fig. 1a). The mean testis weight 30 days after the first immunisation was similar in all investigated animals (Fig. 1a). Histopathological changes and the fibrotic response were assessed by azo-carmine and aniline blue staining (azan), as shown in Fig. 2. The changes in the inflamed testis included reduced diameter of the seminiferous tubules, germ cell sloughing leading to Sertoli cell only tubules, leukocytic infiltrates in the interstitium and thickening of the lamina propria of seminiferous tubules (Fig. 2). Notably, the grade of EAEO development was variable between individual animals, therefore the EAEO groups were subdivided into animals with severe disease symptoms (severe EAEO) and mice showing only mild symptoms of EAEO (low grade EAEO). Testes from the severe EAEO group at 50 (Fig. 2f) and 80 (Fig. 2i) days after the first immunisation represented a complete destruction of testicular morphology with a reduction of the size of the seminiferous epithelium, tubular atrophy and thickening of the seminiferous lamina propria. An increase of the immune cell infiltrates in EAEO testes was observed. In contrast, in the mild form of the disease, some of the seminiferous tubules were still normal. Moreover, the morphological changes were accompanied by a strong fibrotic response in the testes represented by an increase of the collagen fibres around the remaining empty tubules, and in EAEO testes the tunica albuginea was thicker compared to controls (data not shown). Similar changes were also observed in human testicular biopsies with impaired spermatogenesis and inflammatory infiltrates (Supplementary Fig. S1). Moreover, testes from untreated and adjuvant control mice (Fig. 2a,d,g) showed a normal morphology with seminiferous tubules containing Sertoli cells as well as germ cells at all stages of spermatogenesis. At 50 and 80 days after the first immunisation, 100% of animals (7/7 and 5/5, respectively) developed EAEO, whereas in the 30 days EAEO group only 33% (2 of 6) animals showed histological signs of the disease (Fig. 1b). Representative macroscopic difference in testis size between EAEO and control group is shown in Fig. 1c.

### Inflammatory response in EAEO testes

In order to investigate the inflammatory response in the EAEO and control testes, an analysis of different immune cell populations and expression of inflammatory mediators was performed.

### Testicular macrophages and MHC class II molecules are increased in EAEO testes

**Figure 3:**
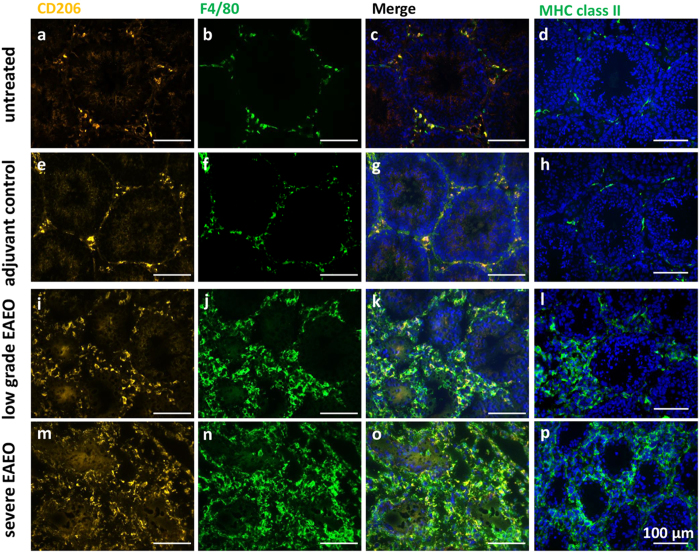
Double staining for CD206 (AlexaFluor546, orange) (**a**,**e**,**i**,**m**) and the macrophage marker F4/80 (AlexaFluor488, green) (**b**,**f**,**j**,**n**) as well as MHC class II single staining (**d**,**h**,**l**,**p**) in testicular cryosections from untreated (**a**–**d**), adjuvant control (**e**–**h**), low grade EAEO (**i**–**l**) and severe EAEO (**m**–**p**) mice. Nuclei were counterstained with DAPI (blue). Under non-inflammatory conditions, co-localized CD206 and F4/80 positive macrophages (**c**,**g**) and MHC class II positive cells (**d**–**h**) were present in low numbers in the interstitial space. An accumulation of double positive F4/80 and CD206 macrophages and MHC class II positive cells was observed in inflamed low grade (**k**,**l**, respectively) and severe (**o**,**p**, respectively) 50 day EAEO testis, with a higher number of F4/80 positive only (CD206-negative) cells. In low grade 50 day EAEO testis (**k**), the accumulation of macrophages was present in areas with reduced tubule diameter, whereas in severe 50 day EAEO testis (**m–p**) macrophages and MHC class II positive cells were more evenly distributed in the interstitial space. Scale bars represent 100 μm.

Although not quantified, immunofluorescence analysis data suggest that the number of cells positive for either F4/80 or CD206, i.e. macrophages, were considerably more prominent in EAEO testes (Fig. 3). In untreated mouse testis, relatively fewer macrophages were found throughout the interstitial space (Fig. 3c). Notably, the majority of these cells were double positive for F4/80 and CD206 indicating that the testicular resident macrophages have an M2 anti-inflammatory phenotype (Fig. 3a,b). A similar distribution of the testicular macrophages was also observed in adjuvant control testis (Fig. 3g). However, it seemed that adjuvant treatment alone led to a slight increase in the number of macrophages as compared to untreated controls. In contrast, in low grade EAEO testis, an accumulation of co-localised F4/80 and CD206-positive cells with increased numbers of F4/80-positive only cells (Fig. 3k) was observed, mainly in the areas where the diameter of the seminiferous tubules was reduced. This accumulation of F4/80-positive only cells was evident in severe EAEO (Fig. 3n) at 50 days after the first immunisation, suggesting that many infiltrating macrophages possess a pro-inflammatory M1 phenotype. In EAEO testis, 30 days after first immunisation, the accumulation of macrophages was less dramatic compared to 50 days EAEO testis and the majority of the macrophage population was double positive for F4/80 and CD206 (Supplementary Fig. S2), indicating an M2-like phenotype. Moreover, the expression of MHC class II molecules responsible for presenting antigenic peptides to CD4+ lymphocytes in normal and inflamed testis was assessed. In normal and adjuvant control testes, MHC class II positive cells were found in low numbers between the tubular basement membrane of adjacent tubules and in the interstitial space (Fig. 3d,h). In contrast, the number of MHC class II positive cells was strongly increased in low grade and severe EAEO testes, mainly in the areas of lymphocytic infiltrates and around damaged seminiferous tubules (Fig. 3l,p). The staining pattern of MHC class II in inflamed testis was very similar to the F4/80 expression pattern (Supplementary Fig. S3). Interestingly, CD206 positive cells in the normal testis did not express MHC class II molecules, whereas in EAEO testes the expression was present (Supplementary Fig. S4). The immunofluorescence data was supported by quantitative RT-PCR showing significantly elevated levels of *H2-Ab1* (MHC class II) mRNA in EAEO testis 50 and 80 days after first immunisation as compared to control animals (Supplementary Fig. S5).

### Increased number of CD45+ leukocytes and CD3+ T cells in EAEO mouse testes

**Figure 4:**
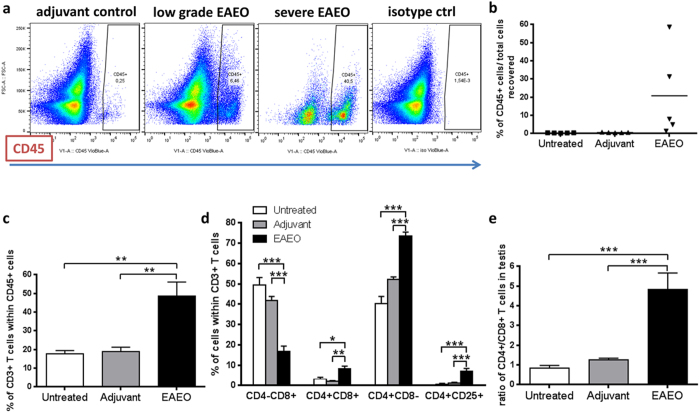
Representative flow cytometry plots for testicular CD45+ leukocytes (**a**) evaluated in the testicular single cell suspension. Percentage of CD45+ leukocytes (**a**,**b**), CD3+ T cells within CD45+ leukocytes (**c**) and different subtypes of CD3+ T cells such as CD4 − CD8+, CD4 + CD8+, CD4 + CD8− and CD4 + CD25+ T cells (**d**) as well as ratio of CD4+/CD8+ T cells (**e**) was analysed in untreated, adjuvant control and EAEO testicular single cell suspension 50 days after the first immunisation, by flow cytometry. After gating out cell debris, doublets and nonviable cells, the population of CD45+ leukocytes and CD3+ T cells was selected for further analysis. Data are expressed as mean ± SEM (n = 5 animals per group); *P < 0.05, **P < 0.01, ***P < 0.001, all other comparisons are not statistically significant.

Flow cytometric analysis revealed an increased percentage of leukocytes (CD45 + ) in cells isolated from EAEO testes (Fig. 4a and b). Depending on the stage of the disease, the highest increase in the number of CD45+ cells was observed in severe EAEO testes, showing in some animals that nearly 50% of testicular interstitial cells were CD45+ leukocytes. Within the population of leukocytes, an increase of CD3+ T cell numbers was observed (Fig. 4c). Further analysis of different T cell subtypes within the gated CD3+ T cell population revealed an increase in the population of CD4 + CD8- and activated CD4 + CD25+ T cells in inflamed testis, while the percentage of CD4 − CD8+ T cells was decreased, as compared to untreated and adjuvant control testes. Interestingly, a new population of double positive CD4 + CD8+ T cells within testicular CD3+ T cell population was identified in EAEO testes (Fig. 4d). Moreover, the CD4+/CD8+ T cell ratio showed approximately 5-fold increase in EAEO testes as compared to untreated and adjuvant control testes (Fig. 4e).

### TNF, MCP-1, and IL-10 mRNA expression is increased in EAEO testes

**Figure 5:**
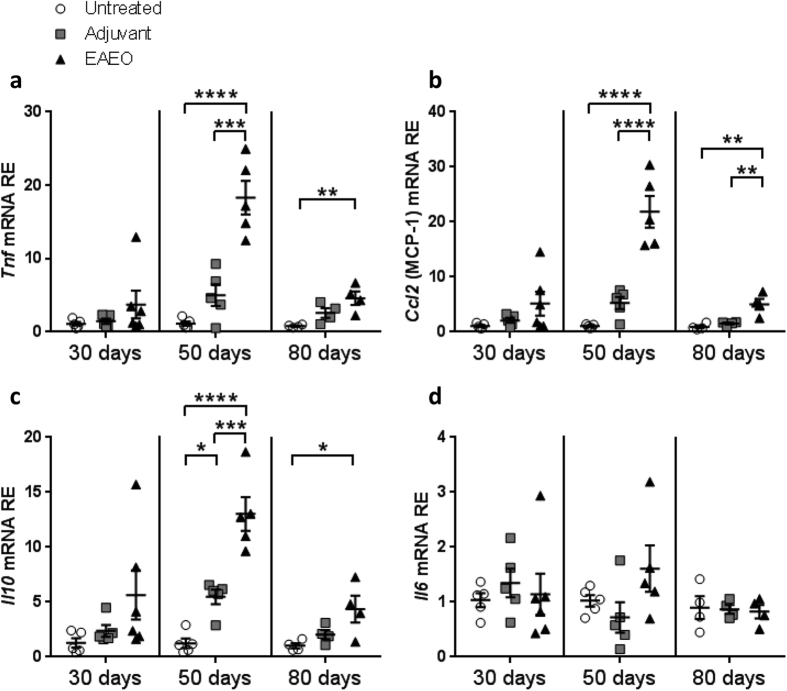
*Tnf* (**a**), *Ccl2* (MCP-1) (**b**), *Il10* (**c**) and *Il6* (**d**) mRNA expression was measured in untreated, adjuvant control and inflamed mouse testis at 30, 50 and 80 days after first immunisation. Relative mRNA levels in mouse testes were analysed using quantitative RT-PCR. Data are represented as mean ± SEM (n = 4–5 animals per group); *P < 0.05, **P < 0.01, ***P < 0.001, ****P < 0.0001, all other comparisons are not statistically significant.

Gene expression of inflammatory mediators was quantified using quantitative RT-PCR. At 30 days after the first immunisation, the mRNA expression levels of TNF, MCP-1 (encoded by the *Ccl2* gene), IL-10 and IL-6 in EAEO were unchanged in all groups (Fig. 5). Further analysis showed an approximately 20-fold increase of TNF (Fig. 5a) and MCP-1 (Fig. 5b) as well as a more than 10-fold upregulation of IL-10 (Fig. 5c) mRNA expression in EAEO testis 50 days after the first immunisation compared to untreated and adjuvant control testes. At 80 days after the first immunisation, an increase of TNF (Fig. 5a), MCP-1 (Fig. 5b) and IL-10 (Fig. 5c) mRNA levels was also observed in EAEO testes as compared to untreated control groups and both untreated and adjuvant controls for MCP-1 (Fig. 5b). Of note, the IL-10 mRNA level was slightly increased in adjuvant control testes as compared to untreated control group 50 days after the first immunisation (Fig. 5c). IL-6 mRNA levels in EAEO were comparable to both controls at all investigated time points (Fig. 5d).

### Altered distribution of α-smooth muscle actin (αSMA) in testis from EAEO mice

**Figure 6:**
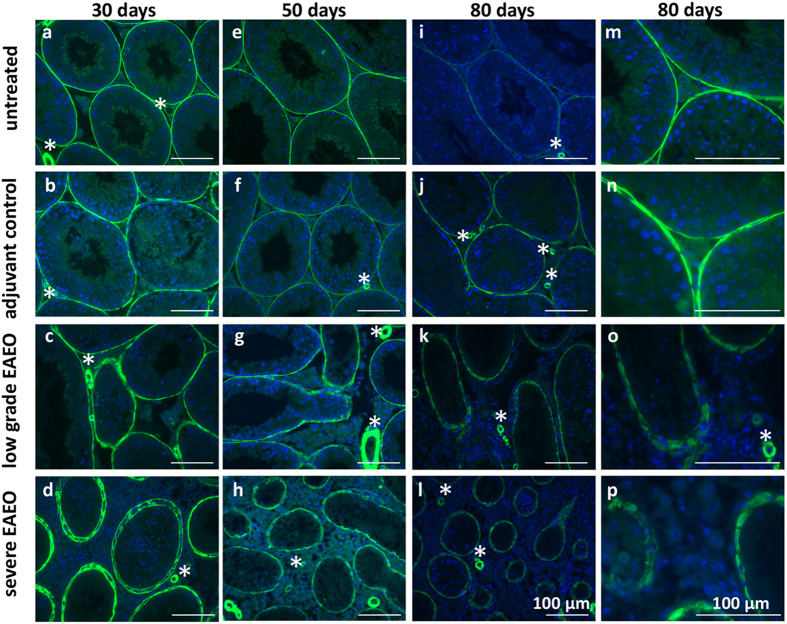
Distribution of α-smooth muscle actin (αSMA) in paraffin sections from untreated (**a**,**e**,**i**,**m**), adjuvant control (**b**,**f**,**j**,**n**), low grade (**c**,**g**,**k**,**o**) and severe EAEO (**d**,**h**,**l**,**p**) testis at 30 (**a**–**d**), 50 (**e**–**h**) and 80 (**i**–**l**) days after the first immunisation. Panels m–p represent a higher magnification of images **i**–**l**. In the testis from untreated and adjuvant controls, αSMA is localised in the peritubular cells as a thin layer, but in low grade and severe EAEO testis, the αSMA is diffusely distributed within the cell. αSMA is also seen in the blood vessels (asterisks) within the testis. Scale bars represent 100 μm.

In order to investigate the peritubular fibrotic response observed in EAEO testis, an analysis of α-smooth muscle actin (αSMA) localisation and distribution by immunofluorescence staining was performed (Fig. 6). The staining revealed a change in the distribution of the αSMA layer in low grade EAEO at 30, 50 and 80 days (Fig. 6c,g,k,o) in areas where the seminiferous tubules were smaller and spermatogenesis was disrupted. The altered distribution and thickening of the αSMA layer was more pronounced in severe EAEO at 30, 50 and 80 days (Fig. 6d,h,l,p). The layer of αSMA was diffusely distributed within the peritubular cells in EAEO testes compared to a thin and compact layer in untreated and adjuvant control testes. The same altered distribution of the layer of αSMA was also observed in human testis samples with focal inflammatory lesions and impaired spermatogenesis (Supplementary Fig. S1).

### Localisation of activin βA in normal and inflamed mouse testes

**Figure 7:**
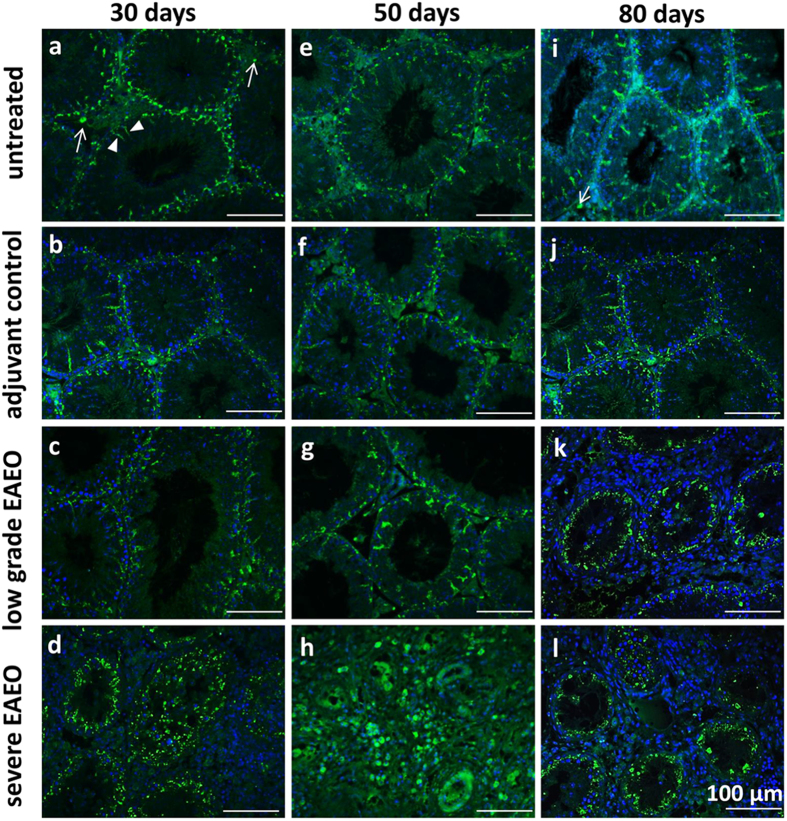
Localisation of activin βA subunit in a testis from EAEO, adjuvant and untreated control mice. Immunofluorescence staining of activin βA subunit using the E4 antibody on paraffin sections from untreated **(a**,**e**,**i**) adjuvant control (**b**,**f**,**j**), low grade (**c**,**g**,**k**) and severe EAEO (**d**,**h**,**l**) at 30 (**a**–**d**), 50 (**e**–**h**) and 80 (**i**–**l**) days after the first immunisation. The activin βA subunit is localised in the cytoplasm of Sertoli cells (arrowheads), peritubular cells and some interstitial cells (arrows) in untreated, adjuvant controls and low grade EAEO. In severe EAEO testis, the staining was present in Sertoli cells and individual immune cells (**d**,**h**,**l**). Scale bars represent 100 μm.

Activin βA immunofluorescence staining (Fig. 7) revealed that the βA subunit was localised mainly in the cytoplasm of Sertoli cells as well as in some interstitial cells in untreated and adjuvant control testes at 30 (Fig. 7a,b), 50 (Fig. 7e,f) and 80 (Fig. 7i,j) days. A similar pattern of activin βA expression was also observed in low grade EAEO at 30 (Fig. 7c), 50 (Fig. 7g) and 80 (Fig. 7k) days. In contrast, in severe EAEO testis at 30 (Fig. 7d), 50 (Fig. 7h) and 80 (Fig. 7l) days after the first immunisation a strong staining of activin βA was detectable in cells within the inflammatory infiltrates and in Sertoli cells.

### Activin A, B, inhibin and activin A receptor expression is changed in mouse EAEO testes

**Figure 8:**
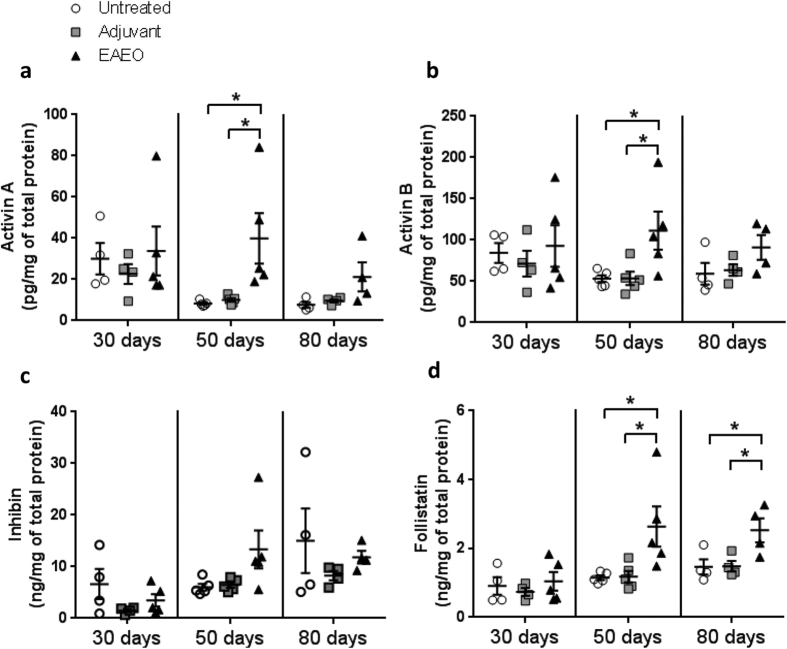
Protein levels of activin A (**a**), activin B (**b**), inhibin (**c**) and follistatin (**d**) were measured in testicular homogenates from untreated, adjuvant control and EAEO animals 30, 50 and 80 days after the first immunisation. Data are represented as mean ± SEM of 4–5 animals per group; *P < 0.05, all other comparisons are not statistically significant.

Activin A (Fig. 8a), activin B (Fig. 8b) and inhibin (Fig. 8c) protein levels in EAEO testes were similar to untreated and adjuvant controls at 30 days after the first immunisation. In contrast, at 50 days EAEO, testes protein levels of activin A and B showed a 4-fold and 2-fold increase, respectively, as compared to untreated and adjuvant control groups. In the chronic phase of the disease at 80 days, no significant change in the testicular concentrations of activin A and B as well as inhibin was measured, although a slight increase in the protein levels of activin A and B was observed as compared to controls (Fig. 8).

**Figure 9:**
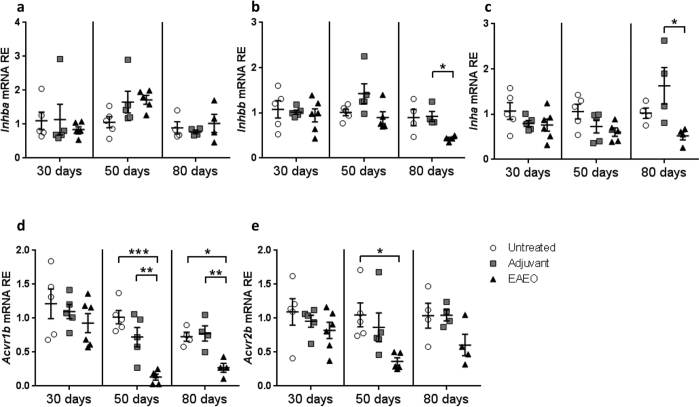
Relative mRNA expression of *Inhba* (inhibin βA) (**a**), *Inhbb* (inhibin βB) (**b**), *Inha* (inhibin α) (**c**), *Acvr1b* (activin receptor, type IB) (**d**) and *Acvr2b* (activin receptor, type IIB) (**e**) in testes from untreated, adjuvant control and EAEO mice 30, 50 and 80 days after the first immunisation analysed by quantitative RT-PCR. Gene expression levels were similar between untreated, adjuvant and EAEO testis at 30 days. Data are represented as mean ± SEM of 4–5 animals per group; *P < 0.05, **P < 0.01, ***P < 0.001, all other comparisons are not statistically significant.

Interestingly, gene expression analysis showed no significant change in activin βA mRNA levels (*Inhba*) in testes from any groups of animals (Fig. 9a). However, the mRNA levels of the activin βB subunit (*Inhbb*) and inhibin α subunit (*Inha*) were decreased in 80 days EAEO testis compared to adjuvant control testes (Fig. 9b and c).

In order to elucidate the influence of testicular inflammatory processes on activin A responsiveness, mRNA expression of activin receptors was analysed. Interestingly, activin A receptor type IB (*Acvr1b*) mRNA expression was decreased at 50 and 80 days, but not at 30 days in EAEO testes compared to untreated and adjuvant control testes (Fig. 9d). Similarly, activin receptor type IIB (*Acvr2b*) mRNA levels were decreased in 50 days EAEO testes compared to untreated control testes (Fig. 9e). No significant difference in the *Acvr2b* mRNA expression levels was detected in testes from 30 and 80 days EAEO animals as compared to control testes (Fig. 9e).

### Follistatin expression is upregulated in EAEO mouse testis

Protein levels of follistatin (Fig. 8d) were unchanged in all groups investigated at 30 days after the first immunisation. In contrast, elevated testicular concentration of follistatin was measured in EAEO at 50 and 80 days after the first immunisation as compared to both controls (Fig. 8d).

The mRNA levels of total follistatin (*Fst*), tissue bound *Fst288* and circulating *Fst315* form of follistatin were not significantly changed in any groups at any time points (Supplementary Fig. S6).

## Discussion

Our results provide evidence that, in a mouse model of testicular inflammation, the levels of testicular activin A, and its less active variant, activin B, are elevated during the course of the disease. Increased levels of activin A were accompanied by upregulated expression of testicular follistatin in the active and chronic stage of EAEO (50 and 80 days, respectively).

In order to mimic the manifestation and symptoms of human testicular inflammation in an animal model and to transfer the model to transgenic animals in the future, we have selected a C57BL/6N mouse strain. EAEO was induced in mice by a modified well-established protocol used previously in rats and mice^6^,^9^,^35^,^36^.

According to the histopathological examination and immune profile of the animals, our observations were in line with the existing data in a mouse and rat model of EAEO, as the most widely used models for studying autoimmune based testicular inflammation^8^,^35^,^36^,^37^. Our results showed the process of development and progression of the disease leading to degeneration of the testicular architecture in late and chronic stage of EAEO in mice at three different time points 30, 50 and 80 days after the first immunisation. At 30 days, only one third of the immunised mice showed histological symptoms of EAEO, while at 50 and 80 days all animals immunised with testicular homogenate in CFA and *pertussis* toxin developed the testicular inflammation characterised by presence of inflammatory infiltrates, disruption of the testicular morphology with a reduction of the diameter of the seminiferous epithelium, sloughing of germ cells and tubular atrophy leading to a smaller testis size. Notably, testicular biopsies from infertile men have been found to contain immunopathologic evidence of focal inflammatory infiltrates and granulomatous orchitis^5^,^38^. Our findings show that the modified immunisation protocol used in this study leads to a reproducible very high induction rate of EAEO in the C57BL/6N mouse strain (100%) compared to previously described studies in different mouse strains (e.g. 89% in C57BL/6N, 68% in B6AF1 or 92% in BALB/cBy strains)^36^,^39^.

The morphological changes in the testis were accompanied by a strong fibrotic response represented by an increase of collagen fibres around the seminiferous tubules. In addition to the collagen deposits, we found a change in the morphological distribution and thickening of the αSMA layer in the peritubular cells. We hypothesise that the inflammatory process in the testis, and subsequent disruption of the testicular morphology, leads to a change of the function of peritubular cells and fibrotic response, as shown previously in human testicular biopsies from patients with impaired spermatogenesis^4^,^40^. As shown by Adam *et al*., the peritubular cells are important players involved in the process leading to fibrotic response in the testis^41^. Notably, very strong formation of collagen deposits was observed in close proximity to the areas with inflammatory infiltrates, granulomas and disturbed testicular morphology. These observations regarding the morphology and fibrotic response suggest that the active phase of EAEO starts after the third immunisation period and the severe form of the disease is reached around 50 days, followed by the chronic form at 80 days. These findings point also to a possible involvement of immune cells in the generation of fibrotic testicular response. Fibrosis is involved in a process of tissue repair after inflammatory induced tissue destruction, however the uncontrolled fibrosis leads to serious health impairment, such as idiopathic pulmonary fibrosis, cirrhosis of the liver or renal failure (reviewed in ref. 12).

In the mouse model of EAEO investigated, an increase of the number of infiltrating leukocytes (CD45+) in the testicular interstitial space was observed, consisting mainly of macrophages and T cells. A similar increase in leukocytic accumulation in inflamed testes has already been described in a rat and murine model of EAEO^36^,^37^,^42^. Interestingly, in the rat EAEO testis, the population of CD4+ and CD8+ T effector cells was increased at the onset of the disease with a predominating population of CD4+ T cells. During the severe phase of the disease, the CD4+ T cell subset decreased, whereas CD8+ T cells were unchanged^43^. In contrast, in our mouse model of EAEO, we observed highly elevated numbers of CD4+ T cells, whereas the population of CD8+ T cells was decreased at 50 days. The data were supported by a higher ratio of CD4+/CD8+ T cells in the testis, which is comparable to many other immune disorders and diseases. In fact, this ratio can also be used as a diagnostic tool for several inflammatory diseases like infectious mononucleosis, chronic lymphocytic leukaemia, Hodgkin disease, anaemia or autoimmune neurological disorder like multiple sclerosis^44^,^45^. In alignment with our data, the evidence for a requirement for the CD4+, but not CD8+ T cell subset for the development of murine EAEO was shown in adoptive transfer experiments^46^. Although the data from testicular biopsies of infertile men is very limited, the inflammatory infiltrates were reported in 4–16% of cases^5^,^47^,^48^. The infiltrating lymphocytes were predominantly identified as CD4+ and CD8+ T cells, which were accompanied by increased numbers of macrophages and mast cells^4^,^49^,^50^,^51^.

Furthermore, a novel finding of the study was the detection of existence of double positive CD4 + CD8+ T cells in the inflamed testis. Initially thought to be exclusively present in the thymus as a developmental stage of T cells, CD4 + CD8+ T cells have been identified recently in other organs^52^. Up to now, the function of these cells in the periphery is not very well investigated and remains controversial. There are reports showing an increase in the number of CD4 + CD8+ T cell subsets in autoimmune and chronic inflammatory disorders, namely in peripheral blood and synovium from patients with rheumatoid arthritis^53^, in fibrotic skin lesions from patients with systemic sclerosis^54^, or in the liver from patients with hepatitis B and C^55^. Further functional studies are necessary for deeper examination of CD4 + CD8+ double positive T cells to fully elucidate their contribution to the immune response and spermatogenic damage in a mouse inflamed testis.

Under normal conditions in a rodent testis, resident macrophages represent a significant population of interstitial cells^56^. Most are resident, anti-inflammatory M2 macrophages that display an immunosuppressive profile, putatively specialised to provide protection for the developing germ cells and involved in maintaining the immune privilege of the testis^57^,^58^. In the present mouse model of EAEO, we have shown that the majority of macrophages in the untreated testis possess an M2 phenotype, as they co-express the F4/80 and CD206 markers, as previously reported by Jaiswal *et al*.^59^. Under inflammatory conditions in later stages of EAEO (50 days), there is an evident increase in the number of testicular macrophages similar as described previously in an EAEO model in BALB/cBy and (C57BL/6 × A/J)F_1_ mice^36^,^60^ and in rats^6^,^7^. Moreover, in human Sertoli cell only and germ cell arrest syndromes, the number of macrophages is typically found to be increased^61^. In contrast, the majority of the F4/80 macrophages in the damaged 50 day EAEO testis do not express the CD206 marker. This finding suggests that the main population of macrophages in EAEO mouse testis consists of pro-inflammatory M1-type macrophages. This is in agreement with several reports showing the inflammatory phenotype and pathogenic role of macrophages in the rat and murine EAEO^7^,^36^,^62^. Interestingly, the majority of macrophages in 30 days EAEO testis seem to possess a regulatory M2-like phenotype. In this light, it is important to mention that testicular activin A could be also involved in the process of the macrophage phenotype determination during inflammatory conditions by inducing transition between M1 and M2 phenotypes, given that M1 are pro-inflammatory and M2 are pro-fibrotic^18^. Moreover, macrophages are involved in the presentation of antigenic peptides by MHC class II molecules, which are recognized by CD4+ T cells^63^. Although the MHC class II expression in normal testis was very low, in inflamed testis the MHC class II positive cells increased dramatically, pointing to a process of autoantigen recognition by T cells. Similar changes have also been observed in B6FA1 mice, showing that approximately 30% of F4/80 positive cells in normal testis were positive for MHC class II molecules, whereas in inflamed testis almost all F4/80 cells were also positive for MHC class II^36^.

Furthermore, TNF, a strong pro-inflammatory cytokine, was increased in our model of EAEO, similar to other mouse models of testicular inflammation induced by viable germ cells^64^ or adoptive transfer by using testis and sperm-antigen specific T cell clones^65^. An increase was detected also in testicular mRNA levels for MCP-1, a potent chemokine, involved in recruiting the immune cells to the site of inflammation. Similar findings were also reported in a rat model of EAEO^8^,^62^. Interestingly, the testicular expression of these mediators was diminished at 80 days of EAEO, but was still elevated as compared to the controls.

Moreover, our study confirms previous findings showing that activin A is located mainly within the Sertoli cells in a normal testis, but it is also present in some interstitial cells; most likely macrophages, as it is known that they produce activin A^66^, and less prominently in the Leydig cells. As a novel finding, our study shows that, under inflammatory conditions, activin A is strongly expressed by Sertoli cells and infiltrating immune cells within the inflamed EAEO testis (50 days). Notably, in the late stage of the disease at 80 days, no significant increase in the levels of activin A either at mRNA or protein level in EAEO testis was observed. Previous reports have shown that activin A levels are elevated in numerous chronic diseases like colitis, meningitis, cancer or autoimmune based disorders like arthritis^25^,^27^,^67^,^68^. We have shown that activin A was also elevated in the present mouse model of autoimmune based testicular inflammation with an increase in the severe form of the disease at 50 days. Our findings suggest that activin A may be involved in the development of autoimmune orchitis, as it has been shown to regulate inflammation and immunity in many tissues^12^. The increase of activin A coincides with the elevated expression of investigated inflammatory mediators, such as TNF or MCP-1.

Moreover, we observed an increase of activin B and inhibin protein levels in the severe form of the disease, but the mRNA levels of the α and βB subunit forming mainly inhibin B were decreased at 80 days. A similar observation was made in a rat model of EAEO demonstrating decreased levels of circulating inhibin B as well as a decreased inhibin α-subunit expression in Sertoli cells^69^.

Curiously, the levels of activin A receptors: ALK4 (*Acvr1b*) and the activin receptor type 2 subunit (*Acvr2b*) in the inflamed testis were significantly lower than normal, while activin A levels were higher. One possible explanation for this observation is that high activin A levels could lead to a negative feedback on the receptor expression levels.

We also showed that the increase of activin A led to a subsequent increase of follistatin expression in the inflamed testis. This is not surprising as it has been shown that activin A can induce expression of follistatin^70^. We suggest that the increase of the follistatin levels may act to counter the effects of activin A during the disease thereby decreasing the severity of the inflammation. Our data demonstrate that higher levels of testicular follistatin at 50 days of EAEO are able to block the increase of activin A expression at 80 days. However the inhibitory function of follistatin at this stage of the disease does not lead to the resolution of inflammation at 80 days. It is likely that the positive effect of follistatin on inhibition of inflammation will be visible at later time points.

## Conclusions

Taken together, our findings point to a pro-inflammatory role for activin A and B in an EAEO mouse model of testicular inflammation. Furthermore, detailed mechanistic and functional studies are necessary to understand the exact role of the activins and their functional antagonists, inhibin and follistatin, in this process leading to severe inflammation causing infertility.

## Materials and Methods

### Animals

Adult 10–12 weeks old C57BL/6N mice were purchased from Charles River Laboratories (Sulzfeld, Germany). Animal experiments were approved by the responsible licensing body of regional ethical committee on animal care (Regierungspraesidium Giessen GI 58/2014 — Nr. 735-GP). All experiments involving animals were carried out in strict accordance with the recommendations in the guide for the Care and Use of Laboratory Animals of the German law of animal welfare. All methods were carried out in accordance with the approved guidelines. The animals were housed in specific pathogen free (SPF) conditions (12 hours light/ dark cycle, 20~22 °C), with access to water and food *ad libitum*.

Mice received analgesia in a form of Tramadol (STADApharm GmbH, Bad Vilbel, Germany) in drinking water (2.5 mg/ml) starting 24 hours before each immunisation and kept for the following 3 days. During immunisation, animals were anaesthetised by inhalation of 3–5% isoflurane. For euthanasia, animals were deeply anaesthetised by inhalation of 5% isoflurane and sacrificed by cervical dislocation.

### Induction of Experimental Autoimmune Epididymo-Orchitis (EAEO)

In order to induce EAEO, adult male C57BL/6 N mice were actively immunised with testicular homogenate in complete Freund’s adjuvant (CFA; Sigma-Aldrich, Saint Louis, USA) as previously described in a rat model^8^, with some modifications. Testicular homogenate (TH) was prepared from decapsulated testes collected from adult syngeneic mice and homogenised in sterile 0.9% NaCl at a ratio of 1:1. Animals were immunised 3 times every 14 days with a mixture of testicular homogenate in CFA at a ratio 1:1, followed by *i.p*. injection of 100 ng *Bordetella pertussis* toxin (Calbiochem, Darmstadt, Germany) in 100 μl Munõz Buffer (25 mM Tris, 0.5 M NaCl, 0.017% Triton X-100, pH 7.6)^37^. Each animal was immunised dorsally by four *s.c.* injections with a total volume of 200 μl (50 μl per injection site).

Adjuvant control animals received CFA mixed with 0.9% NaCl instead of testicular homogenate following the same scheme. Age-matched untreated mice were also included. Animals were sacrificed 30, 50 and 80 days after the first immunisation.

Both testes were removed, weighed, and either snap frozen in liquid nitrogen or fixed in Bouin’s solution for embedding in paraffin. For flow cytometric analysis, fresh testes were used.

### Human testis specimens

Paraffin sections from human testicular biopsies were provided by the Giessen Testicular Biopsy Repository. The specimens had been obtained from infertile men with non-obstructive azoospermia and a histological diagnosis of focal inflammatory lesions associated with disturbed spermatogenesis^4^; biopsies from patients with obstructive azoospermia, i.e. intact spermatogenesis without any signs of inflammation served as control. From all men undergoing testicular biopsy written informed consent was obtained. The study was approved by the local ethics committee of the University Giessen with written informed consent given by all men involved (Ref. No. 100/07). All methods were performed in accordance with the approved guidelines and regulations.

### Histology

Tissue sections (5 μm) from Bouin’s fixed and paraffin-embedded human or mouse testis samples were subjected to routine hematoxylin-eosin and azo-carmine and aniline blue (azan) staining.

### Immunofluorescence staining of testicular macrophages

A double staining of F4/80 (general macrophage marker) and CD206 (a mannose receptor, marker of M2 macrophages) was performed on 8 μm thick testis cryosections fixed in 4% paraformaldehyde (Merck, Darmstadt, Germany) for 10 min, washed and blocked for 1 hour in 10% normal goat serum (Dako, Glostrup, Denmark). Sections were then stained using MaxFluor^TM^ Rat on Mouse Fluorescence Detection Kit (MaxFluor 488) (MaxVision Biosciences Inc., Washington, USA) according to the manufacturer’s protocol. Briefly, sections were incubated in serum-free blocker (MaxVision) for 10 min followed by overnight incubation with rat anti-mouse F4/80 (MCA497G, AbD Serotec, Kidlington, UK) and rabbit anti-mouse CD206 (ab64693, Abcam, Cambridge, UK) antibodies, diluted 1:100 in 1% normal goat serum (Dako, Glostrup, Denmark) at 4 °C. Subsequently, slides were washed and incubated in a rat signal amplifier (MaxVision) for 30 min, followed by MaxFluor488 labelled linker (MaxVision) for 1 hour. Finally, sections were incubated with F(ab′)2-goat anti-rabbit IgG (H + L) AlexaFluor546 (A11071, Life Technologies, Carlsbad, USA) diluted 1:1000 in 1% normal goat serum (Dako, Glostrup, Denmark) for 1 hour at RT. Slides were mounted with ProLong Gold Antifade Mountant with DAPI (Life Technologies, USA).

### Immunofluorescence staining of MHC class II molecules

MHC (major histocompatibility complex) class II staining was performed on 8 μm thick testis cryosections fixed in ice-cold methanol (Sigma-Aldrich, Steinheim, Germany) for 10 min, washed and blocked for 1 hour in 10% normal goat serum (Dako). Sections were incubated overnight with rat anti-mouse MHC class II antibody (clone M5/114.15.2; BioLegend, London, UK) diluted 1:100 in 1% normal goat serum at 4 °C. Subsequently, slides were washed and incubated with donkey anti-rat IgG FITC (Dianova, Hamburg, Germany) diluted 1:200 in 1% normal goat serum for 1 hour at RT. Slides were mounted with ProLong Gold Antifade Mountant with DAPI (Life Technologies).

### Activin A and alpha smooth muscle actin (SMA) staining

Paraffin embedded human or mouse testis (5 μm) sections were boiled in citrate buffer (pH 6.0) for antigen retrieval. Activin A staining was done using the E4 antibody (mouse monoclonal anti-activin βA, Oxford-Brooks University, UK) diluted 1:200 and the MaxFluor^TM^ Mouse on Mouse Fluorescence Detection Kit (MaxFluor 488) (MaxVision Biosciences Inc. Washington, USA) following the manufacturer’s instructions.

α-smooth muscle actin staining was done by incubating sections overnight with the mouse monoclonal FITC conjugated anti-α smooth muscle actin antibody (F3777, Sigma, Saint Louis, USA) diluted 1:1000. Finally slides were mounted with ProLong Gold Antifade Mountant with DAPI (Life Technologies, Carlsbad, USA).

### Preparation of testicular single cell suspension

Decapsulated testes were incubated with 1.2 mg/ml collagenase A and 15 U/ml DNase (Roche Diagnostics, Mannheim, Germany) in PBS in a shaking water bath at 34 °C for 15–30 min. The enzymes were inactivated by adding ice-cold PBS, and the tubule fragments were allowed to settle for 4 min, then the supernatant was filtered and centrifuged at 300 × g for 10 min at 4 °C. The pellet was washed with PBS and erythrocytes depleted by osmotic lysis using red blood cell (RBC) lysis buffer (Qiagen, Hilden, Germany) for 5 min at RT. The final cell suspension was washed in washing buffer (PBS, 0.5% BSA, 2 mM EDTA) at 300 × g for 10 min at 4 °C and processed directly for flow cytometric staining.

### Flow cytometric analysis of T cells

All incubation steps were conducted at 4 °C for 30 min. Briefly, a maximum of 1 × 10^6^ interstitial cells were incubated with mouse FcR blocking reagent (Miltenyi Biotech, Bergisch-Gladbach, Germany) for 10 min at 4 °C. After blocking following monoclonal antibodies (all from Miltenyi Biotec) were used: rat anti-mouse CD45-VioBlue (clone 30F11.1), hamster anti-mouse CD3ɛ-APC (clone 145–2C11), rat anti-mouse CD4-APC-Vio770 (clone GK1.5), rat anti-mouse CD8a-PE-Vio770 (clone 53–6.7) and rat anti-mouse CD25-PE (clone 7D4). Background staining was evaluated using appropriate isotype controls: rat IgG2b-VioBlue, rat IgG2b-APC-Vio770, rat IgG2a-PE-Vio770, rat IgM-PE (all from Miltenyi Biotec) and hamster IgG1-APC (eBioscience, San Diego, USA). Afterwards cells were washed with washing buffer (PBS, 0.5% BSA, 2 mM EDTA). Data were collected for 300,000 events using a MACS Quant Analyzer 10 flow cytometer (Miltenyi Biotech, Bergisch-Gladbach, Germany) and analysed with FlowJo software version 10.0.8 (Ashland, Oregon, USA).

### RNA isolation and quantitative RT-PCR

Total RNA was isolated from frozen mice testis using RNeasy Mini kit (Qiagen, Hilden, Germany) according to the manufacturer’s instructions. On column DNase I treatment (Qiagen, Hilden, Germany) was performed for 30 min at RT to ensure absence of genomic DNA (gDNA) contamination. Reverse transcription was performed with 1 μg RNA sample as described previously^8^. Quantitative RT-PCR was performed using CFX Connect^TM^ real-time PCR detection system (Bio-Rad, Munich, Germany). For the evaluation of *H2-Ab1, Inhba, Inhbb, Inha, Acvr1b, Acvr2b,* total *Fst, Fst288,* and *Fst315* mRNA expression, iTaq Universal SYBR Green Supermix (Bio-Rad) was used. QuantiTect SYBR green PCR Master Mix and QuantiTect primer assay (Qiagen) were used to evaluate *Il6*, *Il10*, *Ccl2* and *Tnf* mRNA expression. All primers used are listed in Supplementary Table S1. Relative gene expression was calculated using the 2^−ΔΔCt^ method^71^ and normalised to three housekeeping genes (β2-microglobulin, HPRT and 18S rRNA) used as internal controls.

### Activin A and B ELISA

Testis samples were homogenised in ice-cold PBS with protease inhibitor cocktail (Sigma) and insoluble components were removed by centrifugation at 14,000 × g for 10 minutes at 4 °C. Total protein measurement was performed using the Pierce BCA Protein Assay (Thermo Scientific, Rockford, USA) following the manufacturer’s instructions. Results were expressed as the amount of protein per mg of total protein. Activin A and B protein levels were measured using ELISA, as described previously^19^,^72^. Sensitivity of activin A ELISA ranged between 6.1 pg/ml and 1.98 ng/ml with an intra-assay coefficient of variability (CV) of 5.6% and the sensitivity of activin B ELISA ranged between 8.1 pg/ml and 2.46 ng/ml and intra-assay CV was 7.8%.

### Follistatin and inhibin measurement by radioimmunoassay (RIA)

Follistatin and inhibin concentrations in mouse testis homogenates were measured using a heterologous, discontinuous RIA, as described previously^14^. Follistatin assay sensitivity ranged between 1.33 and 87.1 ng/ml and an intra assay CV of 9.8%. For the inhibin assay, the intra-assay CV was 6.4% and the sensitivity ranged between 1.24 and 58.76 ng/ml.

### Statistical analysis

Data were expressed as means ± SEM from at least 4 independent animal replicates. Statistical analysis was performed using the one-way ANOVA followed by Tukey’s multiple comparisons when more than 2 experimental groups were compared. P-values < 0.05 were considered as a statistically significant difference. All tests were performed using GraphPad Prism 6 (GraphPad Software, San Diego, USA).

## Additional Information

**How to cite this article**: Nicolas, N. *et al*. Testicular activin and follistatin levels are elevated during the course of experimental autoimmune epididymo-orchitis in mice. *Sci. Rep.* **7**, 42391; doi: 10.1038/srep42391 (2017).

**Publisher's note:** Springer Nature remains neutral with regard to jurisdictional claims in published maps and institutional affiliations.

## Supplementary Material

Supplementary Data
