# Correction: Testicular activin and follistatin levels are elevated during the course of experimental autoimmune epididymo–orchitis in mice

**DOI:** 10.1038/s41598-026-62208-9

**Published:** 2026-07-17

**Authors:** Nour Nicolas, Vera Michel, Sudhanshu Bhushan, Eva Wahle, Susan Hayward, Helen Ludlow, David M. de Kretser, Kate L. Loveland, Hans-Christian Schuppe, Andreas Meinhardt, Mark P. Hedger, Monika Fijak

**Affiliations:** 1 Department of Anatomy and Cell Biology, Justus-Liebig University, Giessen, Germany; 2 Hudson Institute of Medical Research, Melbourne, Victoria Australia; 3 Oxford-Brooks University, Oxford, England; 4 Department of Anatomy and Developmental Biology, Monash University, Melbourne, Victoria Australia; 5 School of Clinical Sciences, Monash University, Melbourne, Victoria Australia; 6 Department of Urology, Pediatric Urology and Andrology, Justus-Liebig University, Giessen, Germany

Correction to: *Scientific Reports* 10.1038/srep42391, published online 13 February 2017

This Article contains errors.

As a result of an error during figure assembly, in the published Figure 7, panel b is a duplication of panel j. This was now revised using the original source data. The correct Figure 7 appears below.

**Fig. 7:**
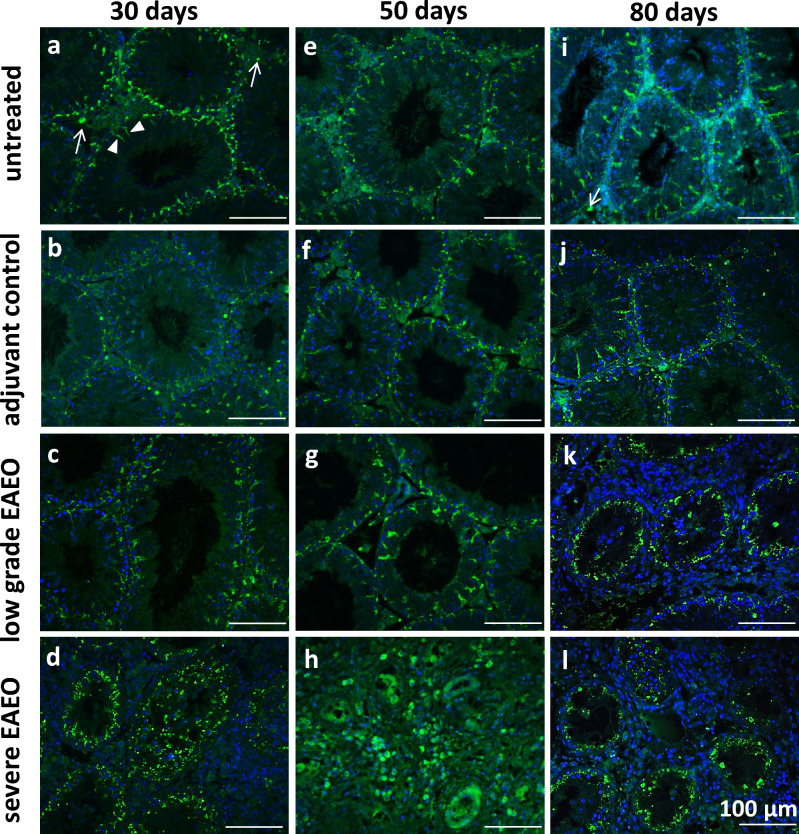
Localisation of activin βA subunit in a testis from EAEO, adjuvant and untreated control mice. Immunofluorescence staining of activin βA subunit using the E4 antibody on paraffin sections from untreated **(a**,**e**,**i**) adjuvant control (**b**,**f**,**j**), low grade (**c**,**g**,**k**) and severe EAEO (**d**,**h**,**l**) at 30 (**a**–**d**), 50 (**e**–**h**) and 80 (**i**–**l**) days after the first immunisation. The activin βA subunit is localised in the cytoplasm of Sertoli cells (arrowheads), peritubular cells and some interstitial cells (arrows) in untreated, adjuvant controls and low grade EAEO. In severe EAEO testis, the staining was present in Sertoli cells and individual immune cells (**d**,**h**,**l**). Scale bars represent 100 μm.

