# Supplementary material for: Testicular activin and follistatin levels are elevated during the course of experimental autoimmune epididymo–orchitis in mice
Source: Sci Rep. 2017 Feb 13;7:42391. doi: 10.1038/srep42391 (PMC5304336; doi:10.1038/srep42391)
Supplement: Supplementary Data [file srep42391-s1.pdf]

Testicular activin and follistatin levels are elevated during the course of experimental autoimmune epididymo–orchitis in mice

Nour Nicolas[1,2], Vera Michel[1], Sudhanshu Bhushan[1], Eva Wahle[1], Susan Hayward[2], Helen Ludlow[5], David M. de Kretser[2,3], Kate L. Loveland[2,4], Hans-Christian Schuppe[6], Andreas Meinhardt[1,2,3], Mark P. Hedger[2,3#] and Monika Fijak[1#*]

[1]Department of Anatomy and Cell Biology, Justus-Liebig University, Giessen, Germany; [2]Hudson Institute of Medical Research, [3]Department of Anatomy and Developmental Biology, [4]School of Clinical Sciences, Monash University, Melbourne, Victoria, Australia, [5]Oxford-Brooks University, Oxford, England, [6]Department of Urology, Pediatric Urology and Andrology, Justus-Liebig University, Giessen, Germany

#Shared last authorship

*Corresponding author: Dr. Monika Fijak, PhD
Department of Anatomy and Cell Biology
Justus-Liebig-University of Giessen
Aulweg 123
D-35385 Giessen, Germany
Phone: +49-641-9947032
Fax: +49-641-9947049
Email: monika.fijak@anatomie.med.uni-giessen.de

Running title: Activin and follistatin in mouse model of EAEO

Key words: experimental autoimmune epididymo-orchitis, testicular inflammation, activin, inhibin, follistatin, fibrosis, immune cells

Supplementary Figure S1

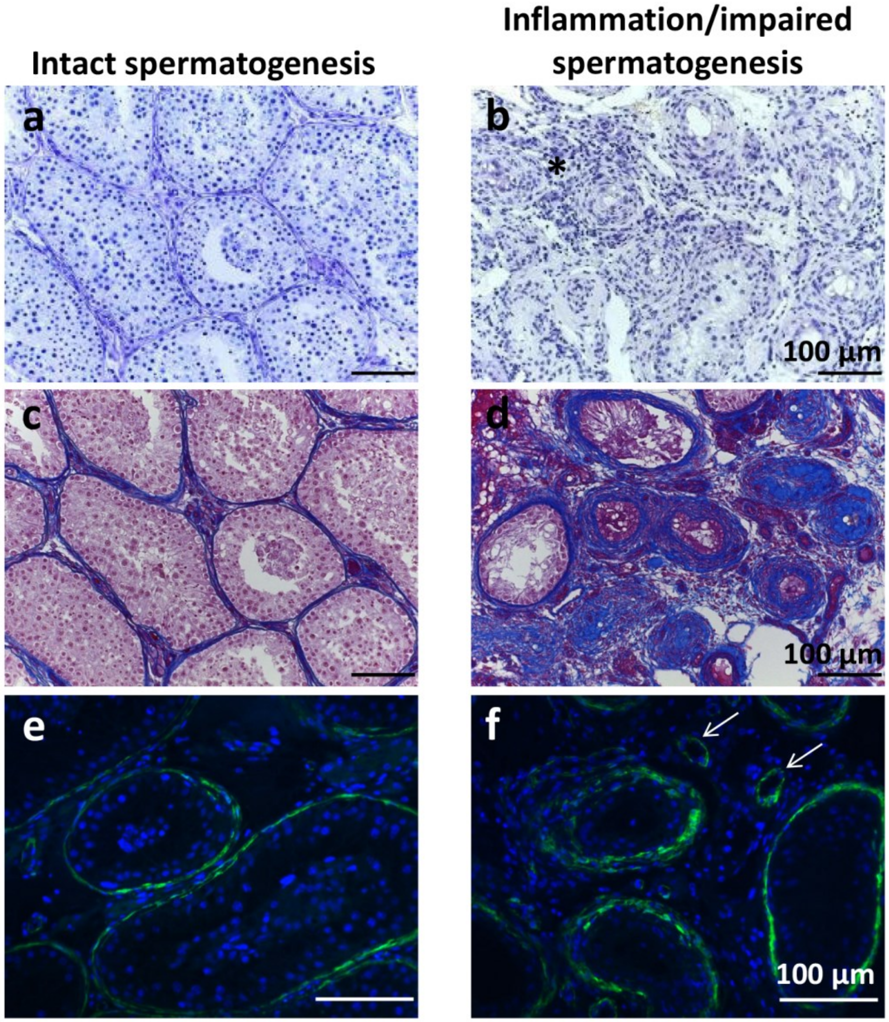

Supplementary Figure S1. Hematoxylin and eosin staining (**a, b**), azo-carmine and aniline blue staining of collagen fibres (**c, d**) and immunofluorescence distribution of αSMA (**e**, **f**) in testicular biopsies from infertile men showing seminiferous tubules with intact spermatogenesis and absence of any signs of inflammation in the interstitial compartment (**a, c**, **e**) and human testis specimens with impaired spermatogenesis and infiltration of non-resident immune cells (**b, d**, **f**). An increase in collagen fibres reflecting severe alteration of the lamina propria (thickening, “meshwork” pattern), tubular atrophy and expanded vascularisation are visible in the testes with impaired spermatogenesis (d) close to the areas with lymphocytic infiltrates (asterisks) (**b**), accompanied by a thicker distribution of the αSMA layer which is spread within the cells (**f**). αSMA is also seen in the blood vessels (arrows) within the interstitium. In testes with normal spermatogenesis, there is no detectable fibrotic response (**c**) and αSMA is localised in the peritubular cells as a thin layer (**e**). Scale bars represent 100 µm.

Supplementary Figure S2.

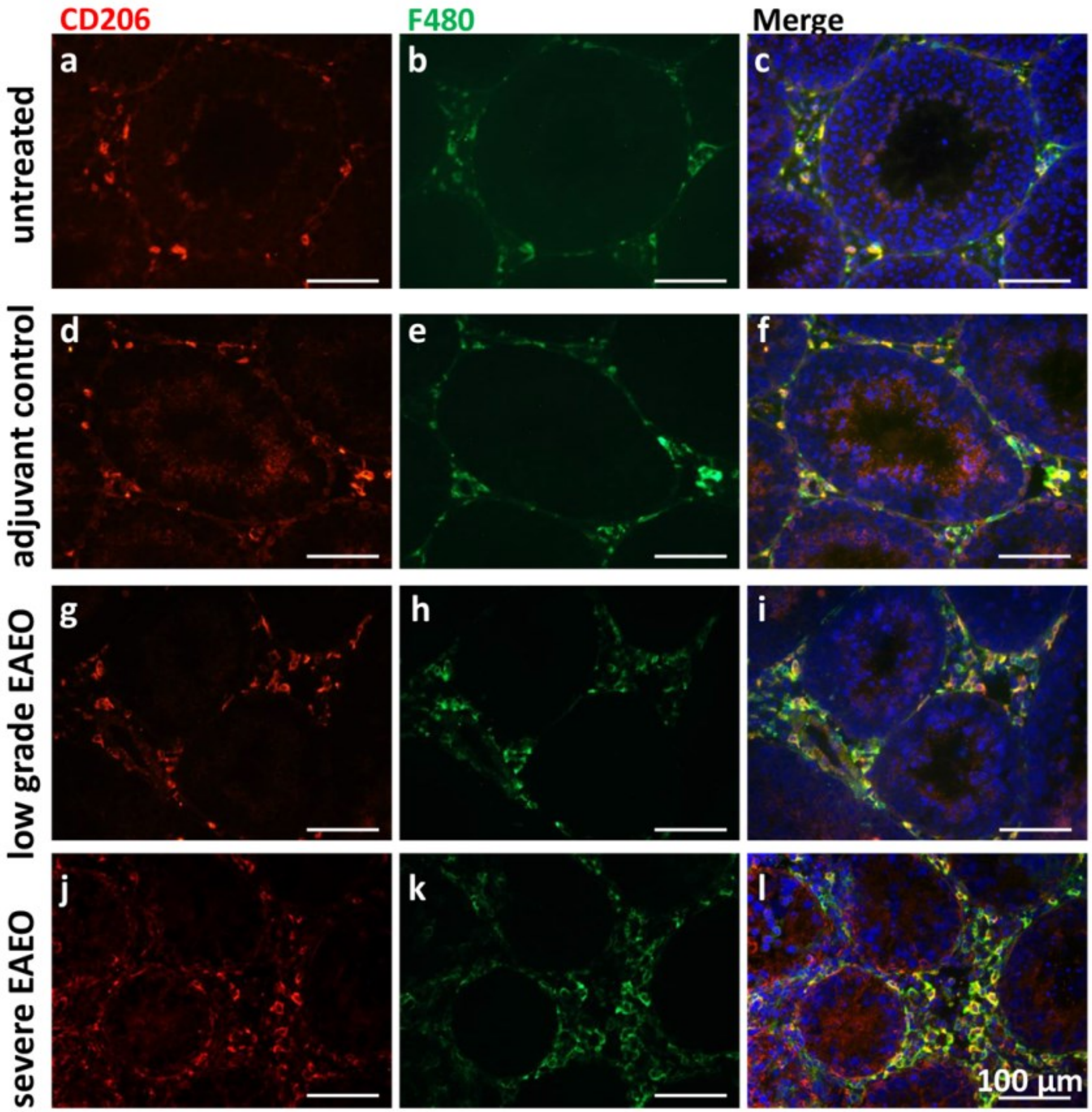

Supplementary Figure S2. Double staining for CD206 (AlexaFluor546, orange) (**a, d, g, j**) and the macrophage marker F4/80 (AlexaFluor488, green) (**b, e, h, k**) in testicular cryosections from untreated (**a - c**), adjuvant controls (**d - f**), low grade EAEO (**g - i**) and severe EAEO (**j - l**) mice 30 days after the first immunisation. Nuclei were counterstained with DAPI (blue). Under non-inflammatory conditions, co-localized CD206 and F4/80 positive macrophages were present in low numbers in the interstitial space (**c, f**). An accumulation of double positive F4/80 and CD206 macrophages was observed in inflamed low grade (**i**) and severe (**l**) EAEO testes. Scale bars represent 100 µm.

Supplementary Figure S3.

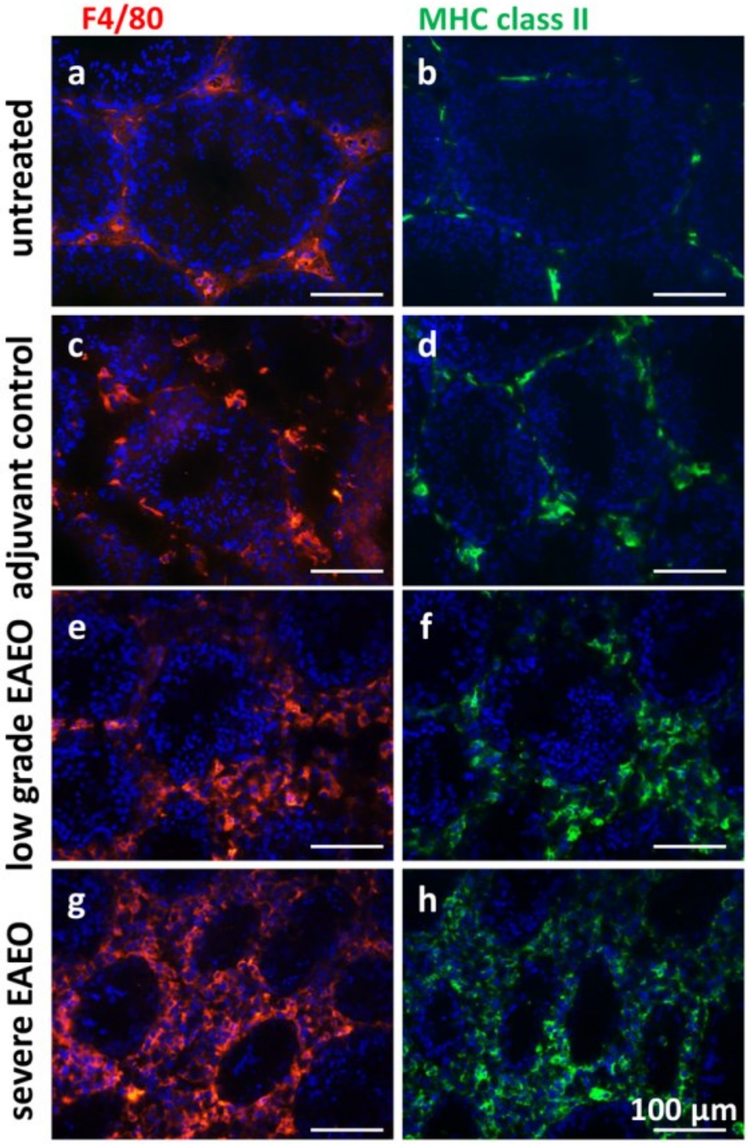

Supplementary Figure S3. Staining for F4/80 (AlexaFluor546, orange) (**a, c, e, g**) and MHC class II molecules (AlexaFluor488, green) (**b, d, f, h**) in testicular cryosections from untreated (**a - b**), adjuvant controls (**c - d**), low grade EAEO (**e - f**) and severe EAEO (**g - h**) mice 50 days after the first immunisation. Nuclei were counterstained with DAPI. In control testis, F4/80 and MHC II positive cells were present in low numbers in in the interstitial space (**a, b, c, d**). An accumulation F4/80 positive macrophages and MHC II positive cells was observed in low grade (**e, f**) and severe (**g, h**) EAEO testes. In low grade EAEO testes the accumulation of F4/80 and MHC class II positive cells was present in areas with reduced tubule diameter (**e, f**), whereas in severe EAEO testes the positive stained cells were more evenly distributed in the interstitial space (**g, h**). Scale bars represent 100 µm.

Supplementary Figure S4.

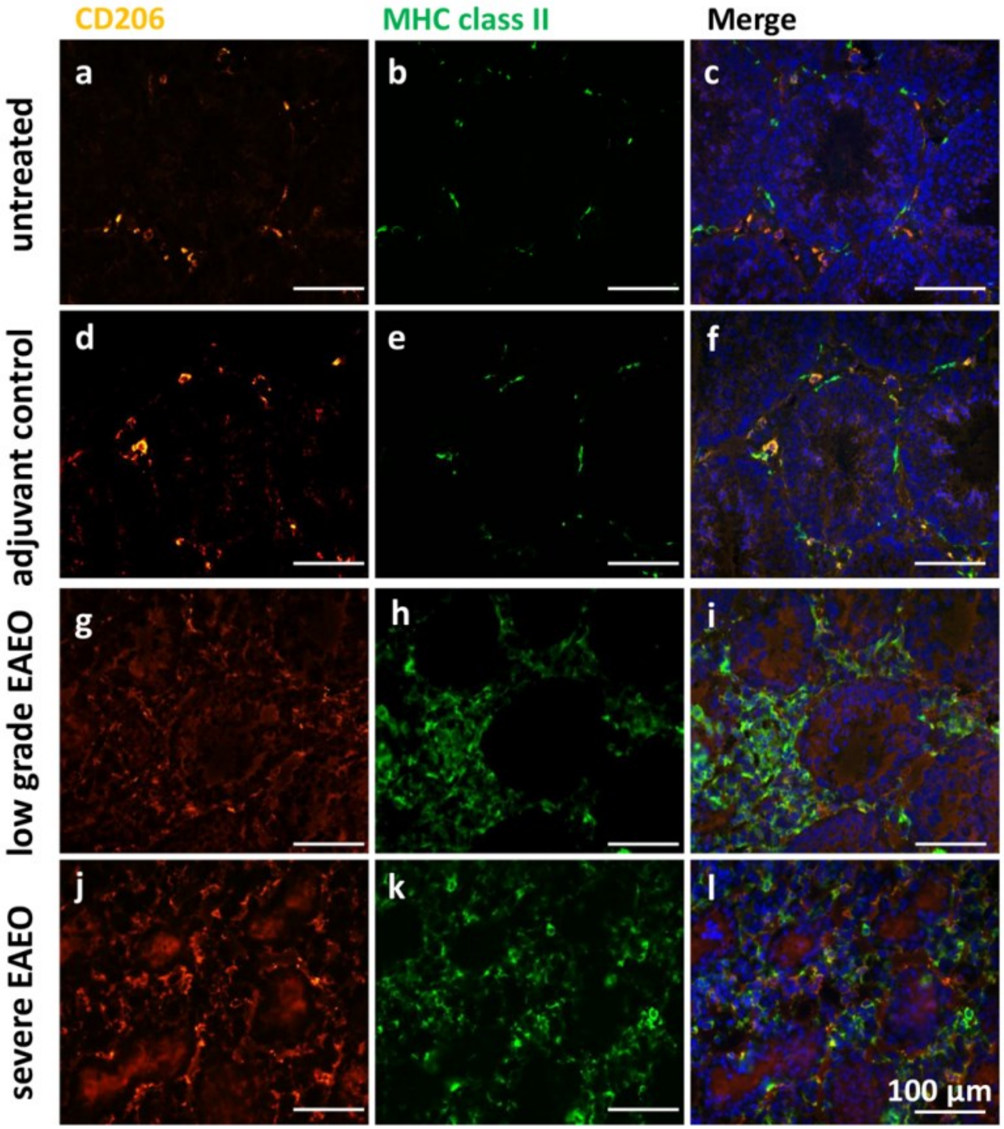

Supplementary Figure S4. Double staining for CD206 (AlexaFluor546, orange) (**a, d, g, j**) and MHC class II (FITC, green) (**b, e, h, k**) in testicular cryosections from untreated (**a - c**), adjuvant controls (**d - f**), low grade EAEO (**g - i**) and severe EAEO (**j - l**) mice 50 days after the first immunisation. Nuclei were counterstained with DAPI (blue). Under normal conditions, rarely co-localized CD206 and MHC class II positive cells were present in low numbers in the interstitial space (**c, f**). An accumulation of CD206 and MHC class II positive cells was observed in inflamed low grade (**i**) and severe (**l**) EAEO testes. In low grade EAEO testes (**i**), the accumulation of positive cells was present in areas with reduced tubule diameter, whereas in severe EAEO testes (**l**) the cells were more evenly distributed in the interstitial space. Scale bars represent 100 µm.

Supplementary Figure S5.

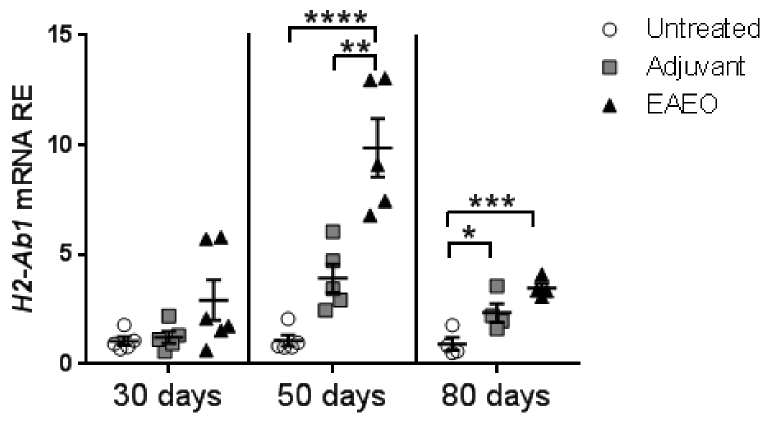

Supplementary Figure S5. Relative mRNA expression of *H2-Ab1* (MHC class II) in untreated, adjuvant controls and EAEO mice testes 30, 50 and 80 days after the first immunisation analysed by quantitative RT-PCR. Data are represented as mean ± SEM of 4 - 6 animals per group; *$p<0.05$, **$p<0.01$, ***$p<0.001$, ****$p<0.0001$.

Supplementary Figure S6.

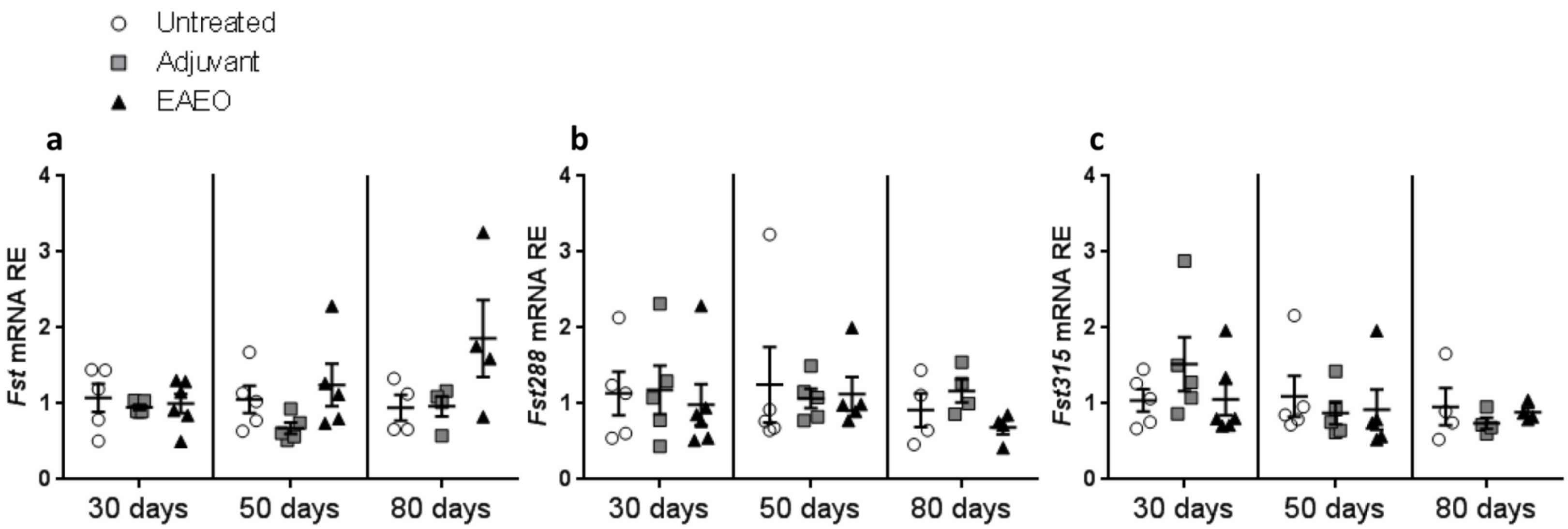

Supplementary Figure S6. Relative mRNA expression of *Fst* (**a**), *Fst288* (**b**), and *Fst315* (**c**) in untreated, adjuvant controls and EAEO mice testes 30, 50 and 80 days after the first immunisation analysed by quantitative RT-PCR. Data are represented as mean ± SEM of 4 - 5 animals per group.

Supplementary Table S1. Primers used in quantitative RT-PCR experiments in this study

Target gene	Forward primer (5'-3')	Reverse primer (5'-3')	Amplicon size (bp)	Annealing temperature (°C)	Entrez Gene ID	Catalogue number (Qiagen)
18S rRNA (*Rn18s*)	TACCACATCCAAGGAAGGCAGCA	TGGAATTACCGCGGCTGCTGGCA	180	55	19791	-
Acvr1b	CCAACTGGTGGCAGAGTTAT	CTGGGACAGAGTCTTCTTGATG	119	55	11479	-
Acvr2b	ATGAGTACATGCTGCCCTTC	CTTAATCGTGGGCCTCATCTT	101	55	11481	-
Β2-microglobulin (*B2m*)	CCGCCTCACATTGAA	TCGATCCCAGTAGACG	198	55	12010	-
Β-actin (*Actb*)	TGACAGGATGCAGAAGGAGAT	TACTCCTGCTTGCTGATCCAC	156	55	11461	-
MCP-1 (*Ccl2*)	QuantiTect Primer assay	Qiagen	118	55	20296	QT00167832
MHC class II (*H2-Ab1)*	AGACGCCGAGTACTGGAACAGCCAGC	CAGAGTGTTGTGGTGGTTGAGGGCCTC	181	60	207105	-
Follistatin (*Fst)*	AGGAGGATGTGAACGACAATAC	CACGTTCTCACACGTTTCTTTAC	95	55	14313	-
Fst288	CTCTCTCTGCGATGAGCTGTGT	GGCTCAGGTTTTACAGGCAGAT	176	55	14313	-
Fst315	CTCTCTCTGCGATGAGCTGTGT	TCTTCCTCCTCCTCCTCTTCCT	192	55	14313	-
HPRT (*Hprt*)	CTGGTAAAAGGACCTC	CTGAAGTACTCATTATAGTCAAG	110	55	15452	-
Il6	QuantiTect Primer assay	Qiagen	128	55	16193	QT00098875
Il10	QuantiTect Primer assay	Qiagen	109	55	16153	QT00106169
Inha	GCCAAGGTGAAGGCTCTATT	AGACCTCCTGTGCATGAAAC	126	55	16322	-
Inhba	AGAACGGGTATGTGGAGATAGA	GACTCGGCAAAGGTGATGAT	97	55	16323	-
Inhbb	CTGCCAGTCGGGCAGGGTATAA	CCTTCACTCCACCAGTCATTT	110	55	16324	-
Tnf	QuantiTect Primer assay	Qiagen	112	55	21926	QT00104006
